# The Effectiveness of Technology-Based Interventions for Reducing Loneliness in Older Adults: A Systematic Review and Meta-Analysis of Randomized Controlled Trials

**DOI:** 10.3389/fpsyg.2021.711030

**Published:** 2021-12-08

**Authors:** Wenjing Jin, Yihong Liu, Shulin Yuan, Ruhai Bai, Xuebin Li, Zhenggang Bai

**Affiliations:** Department of Sociology, School of Public Affairs, Nanjing University of Science and Technology, Nanjing, China

**Keywords:** technology, intervention, loneliness, older adults, meta-analysis

## Abstract

**Objective:** To systematically analyze the effectiveness of technology-based interventions for reducing loneliness in older adults.

**Methods:** We searched relevant electronic databases from inception to April 2021, which included Cochrane Library, PubMed, Web of Science, SpringerLink, EMBASE, CNKI, and Wanfang. The following criteria were used: (i) study design—randomized controlled trial (RCT) designs, (ii) people—older adults (aged ≥ 60 years), (iii) intervention—technology-based interventions in which a core component involved the use of technology to reduce loneliness in older adults; and (iv) outcome—reduction of loneliness level in terms of rating scale scores. Two reviewers independently identified eligible studies, extracted data, and assessed the risk of bias in the included studies. A third reviewer resolved any conflicts. The Cochrane Collaboration's bias assessment tool was used to evaluate the risk of bias for the included studies, and Review Manager 5.4 software was used for the meta-analysis. A random effects model was adopted to measure estimates of loneliness reduction, and standard mean differences (SMD) with a 95% confidence interval (CI) were calculated for each intervention-control contrast, and the I^2^ statistic was applied to examine heterogeneity.

**Results:** A total of 391 participants from six RCTs were included in the review. Of these, three studies were rated as low-quality, and the remaining three were rated as moderate-quality studies. The meta-analysis showed that the evidence regarding the effects on loneliness of technology-based interventions compared with control groups was uncertain, and suggested that technology-based interventions resulted in little to no difference in loneliness reduction compared to control groups (*SMD* = −0.08, 95% CI −0.33 to 0.17, *p* = 0.53). Two types of technology-based interventions were identified: smartphone-based video calls and computer-based training with Internet usage. The subgroup analysis found low-quality evidence to support the effectiveness of both intervention types (*SMD* = −0.01, 95% CI −0.25 to 0.24, *p* = 0.95, and *SMD* = −0.38, 95% CI −0.19, 0.64, *p* = 0.47, respectively).

**Conclusions:** We found no current evidence to support that technology-based interventions were effective compared to different control conditions in reducing loneliness in older adults. This suggests that more research is needed to investigate the effects of technology-based interventions on loneliness in older adults.

## Introduction

Loneliness is usually defined as a subjective negative feeling of a lack of meaningful or intimate social relationships (Gierveld, 1998; Valtorta and Hanratty, 2012). Older adults most commonly experience loneliness in daily life due to their withdrawal from social activities and a reduction of resources available to them (Hind et al., 2014; Fakoya et al., 2020). It is estimated that the global population aged 60 years and over will reach 22% by 2050 (Chen and Schulz, 2016). Therefore, loneliness among the older adult population could be a growing global concern. According to data from the United States and Europe, loneliness was widespread among older adults; ~40% of older adults in these countries experienced some form of loneliness, and this figure could remain constant for the next years (Savikko et al., 2005; Victor et al., 2005; Hind et al., 2014). Victor et al. (2005), for example, examined the prevalence of loneliness among older people in Great Britain, using a self-rating scale. Results showed that approximately one-third (31%) of participants rated themselves as “sometimes” lonely, five percent as “often” lonely, and two percent as “always” lonely. Loneliness is frequently reported by older adults living alone. According to data from China, ~17% of older Chinese adults lived alone in 2011 (Wang and Zhang, 2015). Since the number of older adults living alone is large, the actual number of people experiencing loneliness in China is considerable as well.

The COVID-19 global pandemic has exacerbated the situation. With implementation of social distancing strategies by many countries, millions of older people are confined to their homes or care units. Although social restrictions are crucial to limit the spread of COVID-19, they significantly increase the social isolation of older people, and may result in a severe sense of loneliness within the population group (Wu, 2020; Dahlberg, 2021; Tilburg et al., 2021).

Loneliness can negatively affect various aspects of older adult's daily lives, especially their psychological well-being (Hill et al., 2006; Luanaigh and Lawlor, 2008; Hawkley and Cacioppo, 2010; Hawkley et al., 2010; Nummela et al., 2011). Loneliness is a strong risk factor for low levels of well-being, psychological distress, mental disorders, hopelessness, and depression (Tiikkainen and Heikkinen, 2005; Paul et al., 2006; Golden et al., 2009). Furthermore, loneliness increases mortality rates in older adults and is a predictor of suicide among this population group (Cohen-Mansfield and Perach, 2015).

Loneliness has long been identified as a risk factor for well-being in later life. Over the last few decades, there has been continued interest in developing suitable interventions to alleviate loneliness in older adults. Cattan et al. (2005) conducted a systematic review to summarize and explore the effectiveness of existing interventions for reducing loneliness in older adults. Based on an analysis of 30 research articles that met the review inclusion criteria, the review found that educational and social activity group interventions were most effective for alleviating loneliness in older adults and concluded that more research was needed to evaluate the effectiveness of one-to-one interventions. Whether group interventions or one-to-one interventions identified in the review were face-to-face interventions. In recent years, more intervention types or therapy techniques have been developed to address loneliness in older adults (Sander, 2005; Gardiner et al., 2018; O'Rourke et al., 2018; Poscia et al., 2018). A more recent systematic review conducted by Cohen-Mansfield and Perach (2015) highlighted the value of specific therapy techniques, such as humor therapy, animal aid therapy, and the use of technology in loneliness prevention programs for older adults.

The adoption of technology, especially information and communication technology, to reduce loneliness in older adults has attracted increasing attention worldwide (McCreadie et al., 2002; Magnusson et al., 2004; Ballantyne et al., 2010; Huang, 2010; Choi et al., 2012; Stojanovic et al., 2017; Morton et al., 2018; Pirhonen et al., 2020). Technology-based solutions, for example, the use of video calls and online chat groups, are believed to have potential for maintaining social relations and reducing loneliness during the pandemic (Hwang et al., 2020; Dahlberg, 2021; Tilburg et al., 2021). Although some studies have examined the effects of technology-based interventions on loneliness reduction in older adults, debates about its effectiveness still exist. While some scholars suggest that technologies can increase older adult's interaction with relatives and friends, enabling them to be socially connected without having face-to-face communication, thereby alleviating loneliness (Cattan et al., 2005; Holttum, 2016; Yuan, 2021), other argue that besides the difficulties older adults face when learning to use new technologies, benefits of technology-based interaction could be quite limited, and that increasing online interaction might come at the cost of important face-to-face communication, thus increasing social isolation and loneliness among older adults (Dickinson and Gregor, 2006; Cotten et al., 2013; Helliwell and Huang, 2013).

Considering the controversial opinions about the effects of technology-based interventions for reducing loneliness in older adults, it is necessary to conduct a systematic review to find more reliable evidence. However, to date, few reviews have examined the relationship between technology-based interventions and the reduction of loneliness in older adults. Regarding the research gap in the existing literature, the present study aimed to conduct a systematic review of existing studies that examined the effectiveness of technology-based interventions for reducing loneliness in older adults.

## Methods

### Inclusion and Exclusion Criteria

The inclusion criteria were as follows: (1) study design: randomized controlled trial (RCT) designs; (2) people—older adults (aged ≥ 60 years); (3) intervention—technology-based interventions in which a core component involved the use of technology to reduce loneliness in older adults, and (4) outcome—reduction of loneliness level in terms of rated scale scores. RCTs that had no significant effect on reducing loneliness in older adults were also included and analyzed. The exclusion criteria were as follows: (1) non-Chinese and non-English literature; (2) duplicate studies; (3) incomplete data; (4) full text not available; and (5) studies that included technology-based intervention as a co-intervention.

### Search Strategy

Relevant electronic databases, which included the Cochrane Library, PubMed, Web of Science, SpringerLink, and EMBASE, were searched for eligible studies from inception till April 2021. We also conducted a Chinese language search using the following databases: China National Knowledge Infrastructure and Wanfang. The languages searched were limited to the Chinese and English languages. Electronic searches were performed using various combinations of search terms such as loneliness, technology, computer-based, web-based, smartphone-based, older adults, the elderly, the aged, the seniors, and older adults. For example, using PubMed, the specific search strategy was as follows: (((older people[Title/Abstract] OR older adults[Title/Abstract] OR elderly[Title/Abstract] OR seniors[Title/Abstract] OR 65+[Title/Abstract] OR aged[Title/Abstract] OR “Aged”[Mesh])))° AND (technology[Title/Abstract] OR APP[Title/Abstract] OR software[Title/Abstract] OR web[Title/Abstract] OR Technologies[Title/Abstract] OR smart[Title/Abstract] OR internet[Title/Abstract] OR mobile[Title/Abstract] OR Cell Phones[Title/Abstract] OR computer[Title/Abstract] OR smartphone[Title/Abstract]° OR “Technology”[Mesh])) AND (loneliness[Title/Abstract]° OR “Loneliness”[Mesh]) AND (Randomized Controlled Trial° [Title/Abstract]° OR “Randomized Controlled Trial” [Publication Type]). In addition, we also located articles through references cited in the relevant studies and reviews.

### Selection of Studies

The selection process was conducted in accordance with PRISMA guidelines. Eligibility of studies was determined by two reviewers who independently searched for and selected the literature according to inclusion and exclusion criteria. The reviewers recorded the selection process in detail to complete a PRISMA flow diagram. Any discrepancies between the two reviewers were resolved through discussions with a third reviewer.

### Data Extraction

Two reviewers worked independently to extract trial information. A predetermined data extraction form was used to summarize the characteristics of the included studies, such as information about the articles (e.g., title, author, country), the participants (e.g., gender, age), the intervention and control groups (e.g., intervention techniques, duration of follow-up), and outcome measures (loneliness scale). Any disagreements were resolved by involvement of a third reviewer.

### Outcome Measures

The primary outcome of concern was the changes in loneliness level among older adults with regard to rated scale scores, which were considered the standard mean difference (SMD) with a 95% confidence interval (CI). Outcome measures were determined by the main indicators available in the included studies, such as the University of California Los Angeles Loneliness Scale (Russell et al., 1980) and the De Jong Gierveld Loneliness Scale (Iecovich, 2013; Hind et al., 2014). We analyzed validated self-rated measures if there were no reported clinical-rated measures.

### Assessment of Risk of Bias

Two reviewers independently examined the quality of each eligible trial using the modified Cochrane Collaboration's Tool for Assessment of Risk of Bias. Any disagreements were resolved by a third reviewer. A total of six domains of risk of bias were assessed: selection bias, performance bias, detection bias, attrition bias, reporting bias, and other biases. The risk of bias for each domain was reported as high, low, or unclear. Studies with low risk of bias were considered to be of high quality. The implications of the risk of bias on the results of the included trials are discussed in a later section.

### Data Synthesis

Review Manager 5.4 software was used to generate pooled estimates of effect size. With respect to different loneliness assessment methods, standard mean differences (SMDs) with 95% confidence intervals (CIs) were adopted to analyze the levels of loneliness among older adults. The I^2^ statistic was used to examine heterogeneity across the included studies. Considering the influence of heterogeneity on the results, a random effects model was used to carry out the meta-analysis, as it is a more conservative measure. Subgroup analysis was used to explore the sources of heterogeneity. In the Cochrane Handbook, funnel plots were only suitable for reviews that included more than 10 trials. Therefore, a funnel plot was not generated in this review because only six studies met the inclusion criteria.

## Results

### Literature Screening

**Figure 1:**
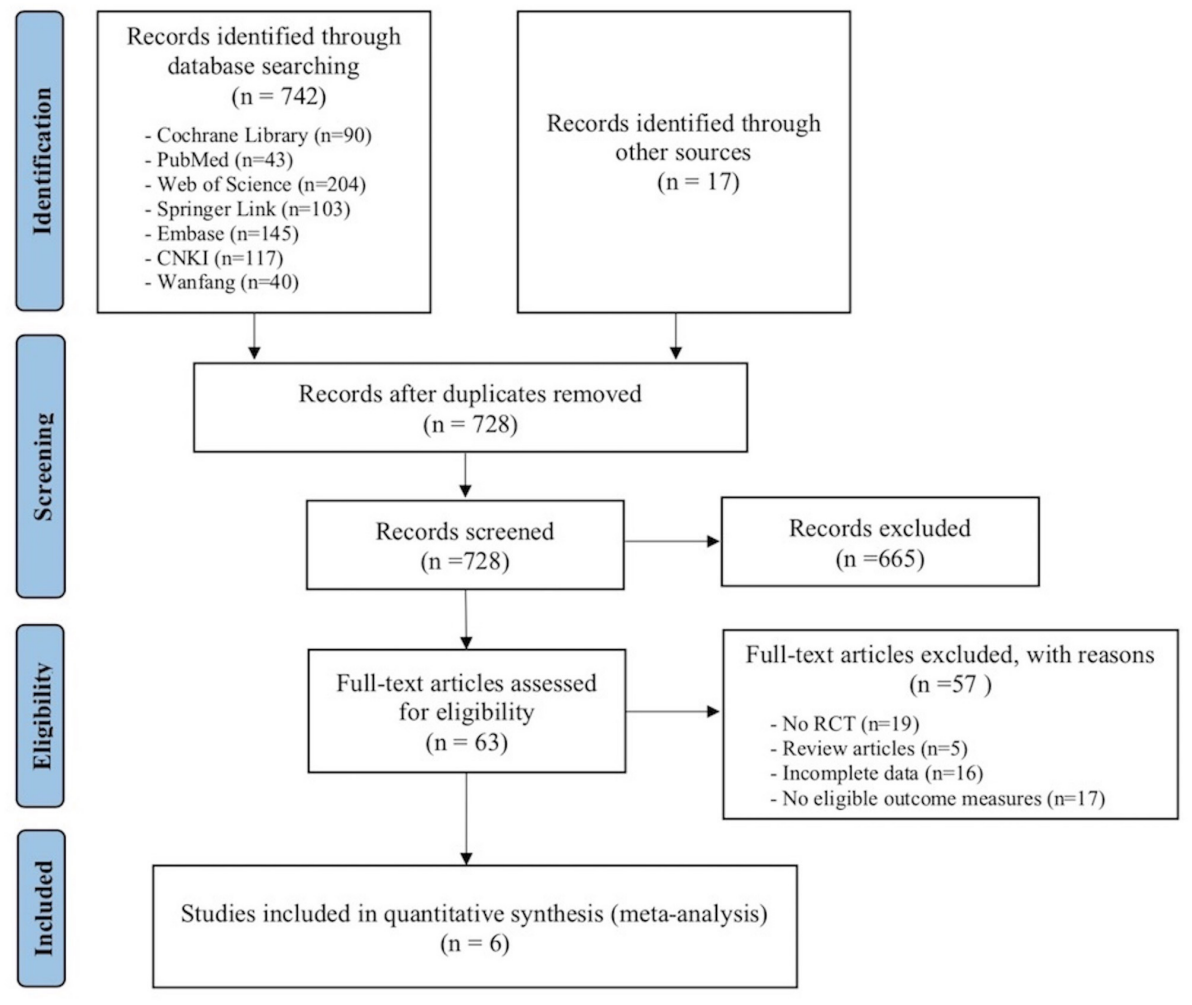
Flowchart of study selection process.

The literature screening resulted in the identification of six RCTs, including a total of 391 participants (Shapira et al., 2007; Slegers et al., 2008; Tsai and Tsai, 2011; Hind et al., 2014; Tsai et al., 2015, 2020). Initially, as shown in Figure 1, a total of 759 studies were identified through a comprehensive literature search. After importing these articles into EndNote X9 software, 31 duplicates were removed. After reading the titles and abstracts of the remaining studies, 665 articles were excluded because they did not meet the inclusion criteria. In addition, 57 articles were excluded after reading their full text, including no RCTs (*n* = 19), review articles (*n* = 5), incomplete data (*n* = 16), and no eligible outcome measures (*n* = 17). The remaining six trials met the eligibility criteria and were included in the review.

### Characteristics of the Included Studies

The characteristics of the included studies are summarized in Table 1. All included studies were RCTs published in peer-reviewed journals. It is appropriate to use partial data from the RCTs included in the meta-analysis. The six RCTs were conducted in Taiwan, China, the United States, Israel, and the United Kingdom between 2006 to 2020. The sample size of the studies ranged from 39 to 95, and the duration of follow-up ranged from three to six months.

**Table 1:** Participant characteristics and study methods of included trials.

**References**	**Country**	**Participants**	**Intervention**	**Comparison**	**Outcome, Tools, and Timing**	**Design**	**Allocation Concealment**	**Blinding**	**Incomplete Data Addressed**	**Free or Selective reporting**
Tsai et al. (2020)	Taiwan, China	Age: 60 and over N: 62	Interact with their family members using a smartphone and a “LINE” application (app).	Control group	Loneliness; the UCLA Scale; three months after baseline.	RCT	Unclear	No	No	Unclear
Tsai et al. (2015)	Taiwan, China	Age: 60 and over N: 49	Receive five min/week of videoconference interaction with their family members for three months.	Regular care	Loneliness; the UCLA Scale; three months after baseline.	RCT	Unclear	No	No	Unclear
Tsai and Tsai (2011)	Taiwan, China	Age: 60 and over N: 90	Use videoconferencing to communicate with their families plus their usual communication activities.	Regular family visits	Loneliness; UCLA Loneliness Scale; three months after baseline.	RCT	Unclear	No	No	Unclear
Slegers et al. (2008)	America	Age: 64–75 N: 56	Three 4-h training sessions over the period of 2 weeks; use the computer freely (once every 2 weeks in the first 4 months, once every month in the remaining period)	Control group	Loneliness; loneliness questionnaire; four months after baseline.	RCT	Unclear	Unclear	Yes	Unclear
Shapira et al. (2007)	Israel	Age: 70–93 N: 95	Computer operation and Internet use (last 15 weeks and included one or two lessons per week).	Taking part in activities like painting and sewing	Loneliness; UCLA Loneliness Scale; 15 weeks after baseline.	RCT	Unclear	Unclear	Yes	Unclear
Hind et al. (2014)	UK	Age: 75 and over N: 39	1-h teleconferences per week for 12 weeks.	Usual health and social care provision	Loneliness; the De Jong Gierveld Loneliness Scale; six months after baseline.	RCT	No	No	Yes	Unclear

*MMSE, mini-mental state examination*.

Each of the six included studies compared technology-based interventions to different interventions and controls: study one (Tsai et al., 2020), two (Tsai et al., 2015) and three (Tsai and Tsai, 2011) examined smartphone-based videoconferencing program and a control group; study four (Slegers et al., 2008) examined computer training, internet usage, and a control group; study five (Shapira et al., 2007) examined computer operation, internet use, and alternative activities; and study six (Hind et al., 2014) examined teleconferences and usual health and social care provision. In terms of intervention protocol, Study one, two, and three required participants in the intervention group to interact with their family members using a smartphone to have a video conference once a week for three or six months. Study four offered three four-h training sessions in two weeks and free computer usage in 12 months. Study five provided one or two lessons per week over a period of 15 weeks, and each lasted ~60 min. Study six conducted a one-to-one volunteer befriender program, providing 10 to 20 min of calls once a week for up to six weeks and one-h teleconferences once a week for 12 weeks.

Study one, two, and three enrolled participants aged 60 and over in nursing homes with basic cognitive abilities and had wireless internet access on their residence floor. Study four enrolled participants aged between 64 and 75 years and excluded older adults who had a cognitive disorder. Studies five and six recruited retired people over 70 years old with mild chronic conditions, who lived independently, and had cognitive functioning. All studies reported on the outcomes of loneliness.

### Quality of the Included Studies

**Figure 2:**
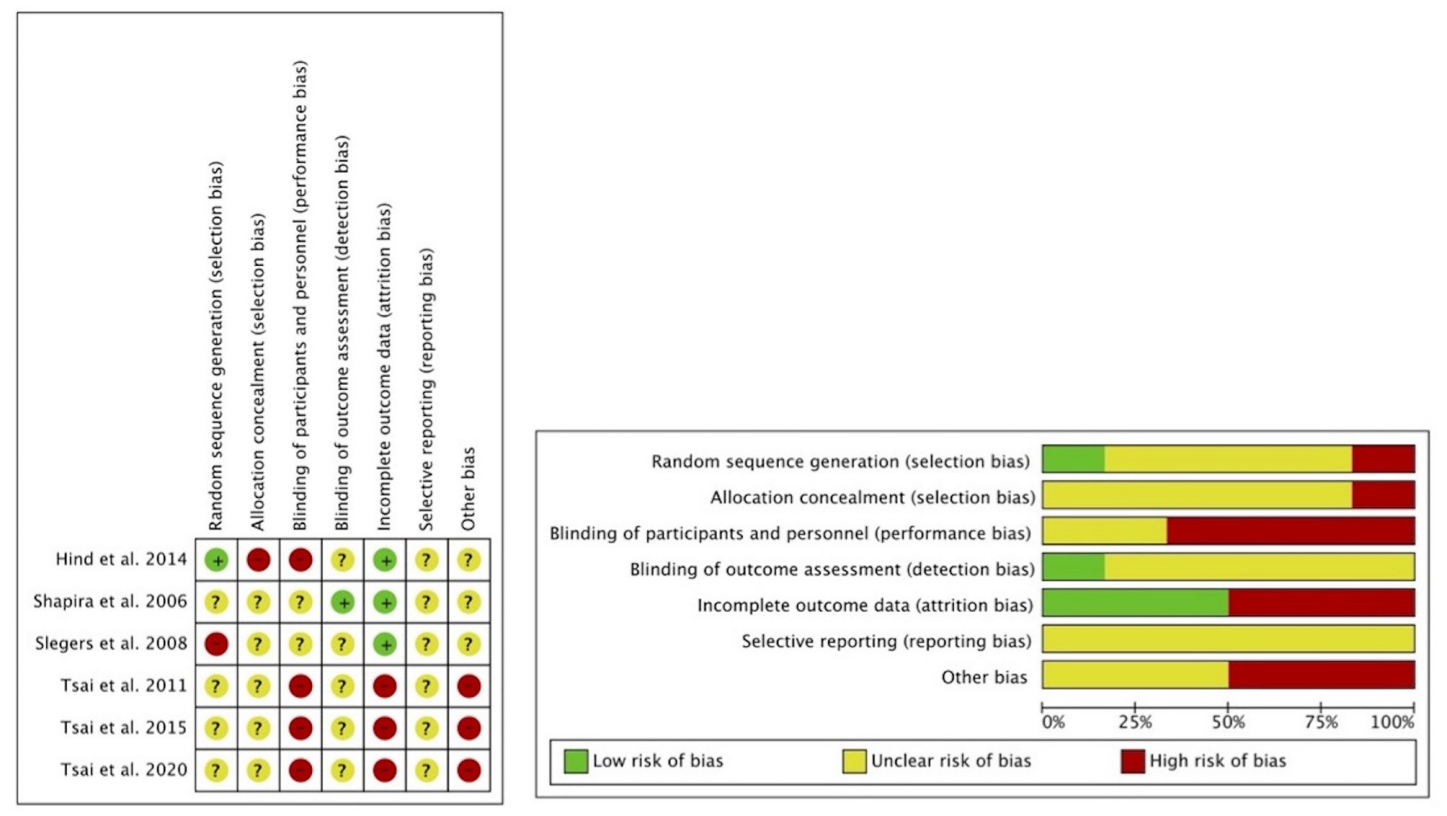
Assessment of risk of bias with selected studies.

Figure 2 shows the assessment of the risk of bias in the six eligible studies. We considered that there was an unclear risk of selection bias and detection bias for three studies, because they lacked information on random sequence generation, allocation concealment, and blinding of outcome assessment (Tsai and Tsai, 2011; Tsai et al., 2015, 2020). Three studies were considered to be at high risk of performance bias because participants were not blinded to the treatment assigned (Tsai and Tsai, 2011; Tsai et al., 2015, 2020). We considered one study to be at a low risk of attrition bias (Slegers et al., 2008) and five studies were at a high risk of attrition bias (Shapira et al., 2007; Tsai and Tsai, 2011; Hind et al., 2014; Tsai et al., 2015, 2020) as a relatively large number of participants in these studies did not complete the training and no data were provided at baseline, follow-up, or both. We considered there to be an unclear risk of reporting bias for all six studies because none of them mentioned the study protocol or analysis intentions, which made it difficult to judge reporting bias. Three studies were judged to be at high risk of other sources of bias (Tsai and Tsai, 2011; Tsai et al., 2015, 2020) because they had similar research designs and were conducted in the same place by the same author; however, the research results showed significant differences.

### Effect of Technology-Based Interventions on Loneliness in Older Adults

**Figure 3:**
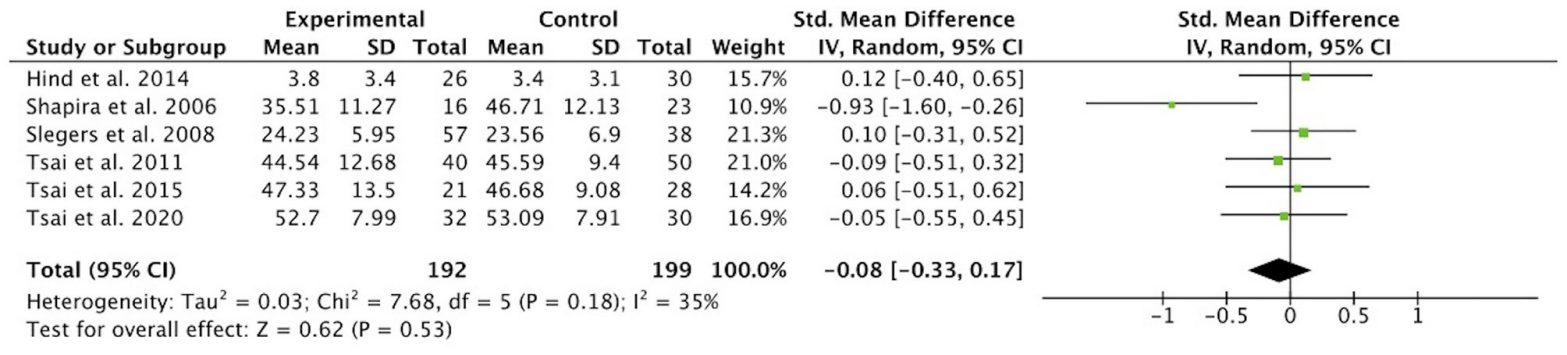
Effect size of technology-based interventions vs. control groups in reducing loneliness rating scores in older adults.

Figure 3 presents the meta-analysis results of the effect of technology-based interventions vs. control groups on loneliness rated scores among older adults. The pooled standardized mean difference (SMD) calculated using the random effects model was −0.08 (95% CI −0.33 to 0.17, *p* = 0.53), which showed that technology-based interventions resulted in little to no difference in reducing loneliness in older adults compared to control groups. Evidence about the effects of technology-based interventions on loneliness reduction among older adults is very uncertain. The value of 35% in the I^2^ statistics reflected moderate heterogeneity. Study one and three showed a reduction in loneliness rated scores in the third month with SMD of −0.05 (95% CI −0.55 to 0.45) and −0.09 (95% CI −0.51 to 0.32) respectively. Study five also found a reduction in loneliness rated scores in the fourth month with an SMD of −0.93 [95% CI −1.60, −0.26]), which suggested no significant differences between technology-based interventions and conventional control. The trials included in this review provided no clear evidence of this effect.

### Subgroup Analysis

**Figure 4:**
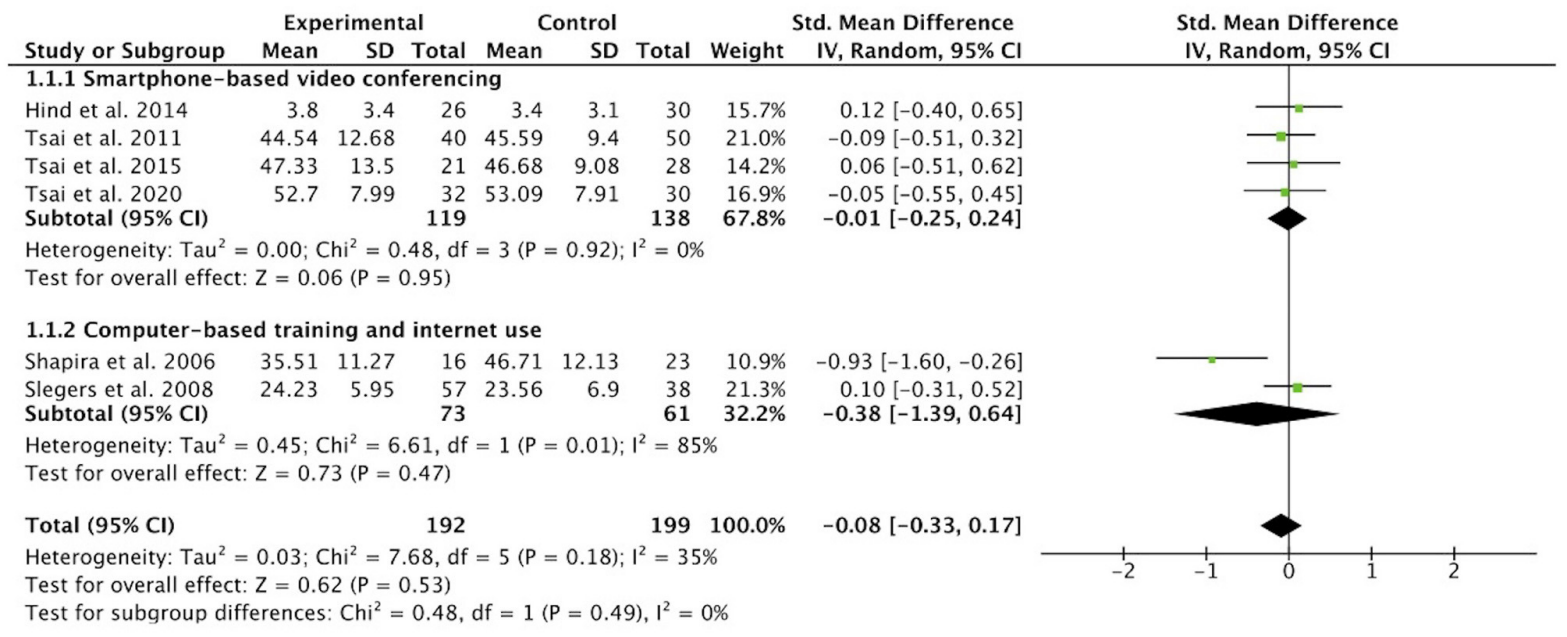
Subgroup analysis: effect size of computer-based interventions or smartphone-based interventions vs. control groups in reducing loneliness in older adults.

Among the six studies included, two studies conducted computer-based training and Internet usage, and four studies used smartphone-based video conferencing. The subgroup analysis examined the effectiveness of each intervention type and found low-quality evidence to support the effectiveness of both intervention types (*SMD* = −0.38, 95% CI −1.39 to 0.64, *p* = 0.47 and *SMD* = −0.01, 95% CI −0.25, 0.24, *p* = 0.95, respectively) (see Figure 4). There was high heterogeneity between studies of computer-based interventions, while there was no heterogeneity between studies of smartphone-based videoconferencing.

Analyses on subgroups, such as age, living conditions, follow-up time, measurement tools, were planned to explore the sources of heterogeneity. There were too few trials and too little information, however, to permit meaningful analyses.

## Discussion

This systematic review is one of the first to address the potential of technology-based interventions to prevent or reduce loneliness, a subjective negative feeling that may deteriorate the physical and psychological well-being of older adults. Six randomized control trials with a total of 391 older participants were assessed to examine the effects of technology-based interventions on loneliness outcomes. All six studies reported loneliness outcomes, making them eligible for the meta-analysis. The results of the meta-analysis showed that technology-based interventions had little or no effect on loneliness reduction in older adults (*SMD* = −0.08, 95% CI −0.33, 0.17, *p* = 0.53). There was a wide variation among the included trials in terms of sample size, participants' demographics, measurement tools, intervention methods, and duration of the intervention. Heterogeneity in the comparison interventions was resolved through subgroup analyses. We used subgroup analysis to examine the effect of different intervention types on loneliness outcomes in older adults. The results of the subgroup analysis showed that the effectiveness of computer-based training and Internet usage and smartphone-based video conferencing on reducing loneliness in older adults were both very uncertain (*SMD* = −0.38, 95% CI −1.39 to 0.64, *p* = 0.47 and *SMD* = −0.01, 95% CI −0.25, 0.24, *p* = 0.95, respectively) when compared with various control groups.

Subgroup analysis for age, living conditions, follow-up time, and measurement tools were planned; however, there were not enough trials or information to perform meaningful analyses. Technology-based interventions were compared with usual activities, no intervention, regular family visits, regular health and care provision, and group activities. However, these comparisons could not be analyzed because most of the alternative interventions were part of a single study.

We only included six studies in the review because few studies have focused on the effect of technology-based interventions on loneliness outcomes for older adults. Among these studies, some only collected baseline data, and some did not present data on loneliness outcomes, so they were excluded from this review. Among the six included studies, study one, two, and three were rated as low-quality studies as they were assessed to have three high bias risk and none of the low bias risk according to Cochrane Collaboration's Tool for Assessment Risk of Bias. The remaining three studies were rated as moderate-quality studies, as they were assessed to have three or more unclear bias risks. Quality issues mainly included imprecision and the risk of bias.

By introducing electronic devices to older adults, technology-based interventions are designed to help increase social connections and reduce loneliness. However, the results of the present review showed that technology-based interventions could be ineffective in alleviating loneliness in older adults. Our review did not support the conclusion of a review published in 2015 that showed that technology-based interventions were effective for alleviating loneliness in both one-to-one and group formats, as well as in samples of community residents and institutionalized persons (Cohen-Mansfield and Perach, 2015). The overall evidence about the effectiveness of technology-based interventions for reducing loneliness in older adults is mixed, and results from existing reviews and individual clinical trials vary. The diversity of interventions involving dose, duration, location, and intervention types, along with methodological constraints such as small samples and various risk of bias, may account for the disparate results.

Based on the meta-analysis and a systematic review of existing literature, we identified some key issues that may be helpful for examining the true effects of technology-based interventions on loneliness outcomes in older adults. First, regarding research participants, we noted that heterogeneity among older adults was largely neglected in existing literature. According to Hind et al. (2014), loneliness does not progress linearly across old age; instead, it peaks among the oldest-old who are aged 80 and over. Oldest-old individuals are more likely to experience loneliness and social isolation when compared with younger individuals. Technology-based interventions could be more helpful and beneficial for those who experience loneliness. Future studies or reviews should focus on older adults who have a high risk of experiencing loneliness in daily life, such as the oldest-old group, especially those who are homebound. Second, regarding intervention methods, we noted that the main intervention methods were smartphone-based videocalls, computer-based training and Internet usage. Exploring technology-based interventions more broadly may help to identify more evidence on the role of technology in reducing loneliness in older adults. Third, in terms of study design, we noted that studies that adopted an RCT design were quite limited. Including a wider range of study designs may help to find more potentially informative evidence. Fourth, most studies had no or limited follow-up which makes it difficult to examine the ongoing effect of technology-based interventions on loneliness outcomes. Hence, studies over a longer time period should be undertaken. Finally, more high-quality research is required on the effects of technology-based interventions on loneliness outcomes among older adults.

This systematic review has some important limitations. First, the included studies did not specifically target older adults who were demonstrably lonely to determine the effects of technology-based interventions on loneliness outcomes among them. The second limitation was the small sample size employed in several studies. The third was that the number of studies included in the review was relatively small, which may limit our understanding of the overall effects of technology-based interventions on loneliness outcomes among older adults and effects under different conditions. Finally, the present systematic review only concentrated on loneliness in older adults, and other meaningful indicators, such as depression, social support, and quality of life, were not analyzed, which limited the examination of the overall effects of technology-based interventions.

## Conclusions

The present systematic review concluded that evidence on the effectiveness of technology-based interventions for alleviating loneliness in older adults is uncertain. Although the results of the present review showed that technology-based interventions had little to no differences in alleviating loneliness in older adults when compared with control groups, this does not mean that this kind of intervention is absolutely ineffective in clinical practice. According to existing research, technology usage, at a minimum, has no harmful effects on older adults who experience loneliness and desire social connection. For practical purposes, caution should be given to older adult's abilities, conditions, needs, and resources to determine whether technology-based interventions are an appropriate approach for reducing loneliness. Essentially, technology was not a solution to reduce loneliness in older adults, but a tool that helps them stay connected with their family members, friends, neighbors, etc., and get access to information and resources. We argue that more research should be conducted to explore the mechanisms of technology-based interventions to alleviate loneliness in older adults in the future.

## Data Availability Statement

The original contributions presented in the study are included in the article, further inquiries can be directed to the corresponding author/s.

## Author Contributions

WJ designed the study. WJ, YL, and SY performed the literature search, article selection, quality appraisal, statistical analysis, and wrote the first draft of the manuscript. ZB supervised the paper production. WJ, ZB, RB, and XL participated in the revision of the subsequent draft. All authors read and approved the final manuscript.

## Funding

This study was financially supported by the MOE (Ministry of Education in China) Project of Humanities and Social Sciences (Grant No. 19YJCZH001). The funder had no role in the study design, paper writing, or decision to publish.

## Conflict of Interest

The authors declare that the research was conducted in the absence of any commercial or financial relationships that could be construed as a potential conflict of interest.

## Publisher's Note

All claims expressed in this article are solely those of the authors and do not necessarily represent those of their affiliated organizations, or those of the publisher, the editors and the reviewers. Any product that may be evaluated in this article, or claim that may be made by its manufacturer, is not guaranteed or endorsed by the publisher.
